# Thirteenth District Medical Society

**Published:** 1854-06

**Authors:** James Beck


*Thirteenth District Medical Society, Indiana.*

DEAR SIR: I thought it my duty to drop a few lines to you, to let you know that we are still punctual in holding our Medical Meetings first Monday of May every year.

The Thirteenth Medical District Society of Indiana, which is the oldest and one among the most respectable societies in the State, held their 24th annual Meeting in New Castle, Indiana, May 1st, 1854; and being the time of holding their election, a revision of the former bill of prices was adopted, with an addition of twelve per cent. The bill of prices occupied nearly the entire meeting.

Dr. J. W. Lynch, a graduate of Rush Medical College, was admitted a member, after which the following officers were elected for the ensuing year, viz:

*President*—John C. Beck, M. D.
*Secretary*—John W. Lynch, M. D.
*Treasurer*—Dr. John Parr.
*Censors*—A. Jackson Batson, M. D., Dr. Jas. Beck, Joel Reed, M. D.

This Society is in a flourishing condition and bids fair to have a long and prosperous life. Good luck to our *Alumni.*

James Beck, M. D.

